# New Thiazolyl-triazole Schiff Bases: Synthesis and Evaluation of the Anti-*Candida* Potential

**DOI:** 10.3390/molecules21111595

**Published:** 2016-11-22

**Authors:** Anca Stana, Alexandra Enache, Dan Cristian Vodnar, Cristina Nastasă, Daniela Benedec, Ioana Ionuț, Cezar Login, Gabriel Marc, Ovidiu Oniga, Brîndușa Tiperciuc

**Affiliations:** 1 Department of Pharmaceutical Chemistry, “Iuliu Hațieganu” University of Medicine and Pharmacy, 41 Victor Babeș Street, RO-400012 Cluj-Napoca, Romania; teodora_anca@yahoo.com (A.S.); ale.enache@yahoo.de (A.E.); ionut.ioana@umfcluj.ro (I.I.); marc.gabriel@umfcluj.ro (G.M.); onigao65@yahoo.com (O.O.); brandu32@yahoo.com (B.T.); 2 Department of Food Science, University of Agricultural Sciences and Veterinary Medicine, 3-5 Mănăștur Street, RO-400372 Cluj-Napoca, Romania; dan.vodnar@usamvcluj.ro; 3 Department of Pharmacognosy, “Iuliu Hațieganu” University of Medicine and Pharmacy, 12 Ion Creangă Street, RO-400010 Cluj-Napoca, Romania; dani_67ro@yahoo.com; 4 Department of Physiology, “Iuliu Hațieganu” University of Medicine and Pharmacy, 1 Clinicilor Street, RO-400006 Cluj-Napoca, Romania; cezar.login@umfcluj.ro

**Keywords:** Schiff base, triazole, fungicidal activity, docking, ADME

## Abstract

In the context of the dangerous phenomenon of fungal resistance to the available therapies, we present here the chemical synthesis of a new series of thiazolyl-triazole Schiff bases **B1**–**B15**, which were in vitro assessed for their anti-*Candida* potential. Compound **B10** was found to be more potent against *Candida* spp. when compared with the reference drugs Fluconazole and Ketoconazole. A docking study of the newly synthesized Schiff bases was performed, and results showed good binding affinity in the active site of co-crystallized Itraconazole-lanosterol 14α-demethylase isolated from *Saccharomyces cerevisiae*. An in silico ADMET (absorption, distribution, metabolism, excretion, toxicity) study was done in order to predict some pharmacokinetic and pharmacotoxicological properties. The Schiff bases showed good drug-like properties. The results of in vitro anti-*Candida* activity, a docking study and ADMET prediction revealed that the newly synthesized compounds have potential anti-*Candida* activity and evidenced the most active derivative, **B10**, which can be further optimized as a lead compound.

## 1. Introduction

Over the last years, invasive fungal infections caused by *Candida* have become a major cause of morbidity and mortality in immunocompromised patients. In clinic, the number of antifungal agents that are available is limited. These drugs belong to major classes of azoles, allylamines, polyenes, fluropyrimidines, and thiocarbamates. The azole molecules are the most widely used [1]. They act by inhibiting CYP51 (lanosterol-14α-demethylase), an enzyme that catalyzes the demethylation of lanosterol into ergosterol. Due to the inhibition, the steroid accumulates and alters the permeability and rigidity of plasma membranes. Azole antifungal agents inhibit the enzyme by forming a bond between the heterocyclic nitrogen atom (N-3 of imidazole and N-4 of triazole) and the heme iron atom in the binding site of CYP51. Because of the existence of CYP51 in fungi and mammals and because of the effects of these compounds on CYP3A4 (Cytochrome P450 3A4), the selective inhibition of fungal CYP51 is very important and results in an increased therapeutic index [2].

Unfortunately, the extensive use of azole molecules as antifungals has led to the appearance of severe fungal resistance. The majority of candidiasis is caused by *Candida albicans*, followed by *C. glabrata*, *C. tropicalis*, *C. parapsilosis* and *C. krusei*. Some types of *Candida* became resistant to the first and second line of antifungal treatment (fluconazole, echinocandins). Most of the isolated strains are of *C. glabrata*. A growing concern worldwide is represented by the multidrug-resistant *Candida* infections, which no longer respond to the treatment with fluconazole or echinocandins. The number of drugs available is surpassed by the large spread of fungal infections. Furthermore, they present a series of disadvantages, such as reduced bioavailability, marked hepatotoxicity and many drug interactions, which limit their use. Due to the facts presented above, the resistance and the toxicity of available antifungals, and the development of new antifungal molecules, which can act by a different mechanism of action, have a better pharmacokinetic and safety profile and are efficient against the resistant strains, are urgently required.

Schiff bases, key intermediates in organic synthesis and also as common ligands in the coordination chemistry [3], have been shown to exhibit a broad range of biological activities, including antifungal, antibacterial [4], antiviral, antihelmintic, antiproliferative [5], antioxidant [6]. The imine group present in such compounds has been shown to be essential for their biological activities [7]. Thiazoles, triazoles and their derivatives play an important part in heterocyclic chemistry due to their biological activity [8,9,10]. Fluconazole and voriconazole, broad spectrum antifungals, contain these heterocyclic systems incorporated into their structures. The ample evidence reported in the literature on the biological potential of Schiff bases containing thiazole and triazole moieties in their structure [11] led us to the synthesis, physico-chemical characterization and antifungal evaluation of new Schiff bases containing both heterocycles.

The pharmacokinetic and pharmacodynamic behavior of molecules inside the human body is influenced by their molecular properties, molecular size, flexibility and the presence of different pharmacophore features. Almost one half of the drug failures in development phases are due to suboptimal pharmacokinetic properties and unacceptable toxicity [12]. The in vivo experimental determination of pharmacokinetic parameters of newly synthesized compounds is uneconomical and time consuming. From this point of view, computational methods which can predict the ADMET (absorption, distribution, metabolism, excretion, toxicity) properties of the new compounds could help to eliminate the molecules likely to fail in the early stage of drug discovery. In order to predict some pharmacokinetic and pharmacotoxicological properties, an in silico ADME-Tox (absorption, distribution, metabolism, excretion, toxicity) study was realized on the newly synthesized compounds. 

Docking studies are useful in drug discovery to predict the docked structure of the ligandreceptor complex and to rank the ligand molecules based on their binding energy. Docking protocols help elucidating the most energetically favorable binding pose of a ligand to its receptor. The new derivatives were docked into the active site of lanosterol-14α-demethylase from *Saccharomyces cerevisiae*, to facilitate the understanding of the antifungal mechanism of action of the synthesized Schiff bases.

## 2. Results and Discussion

### 2.1. Chemistry

**Scheme 1:**
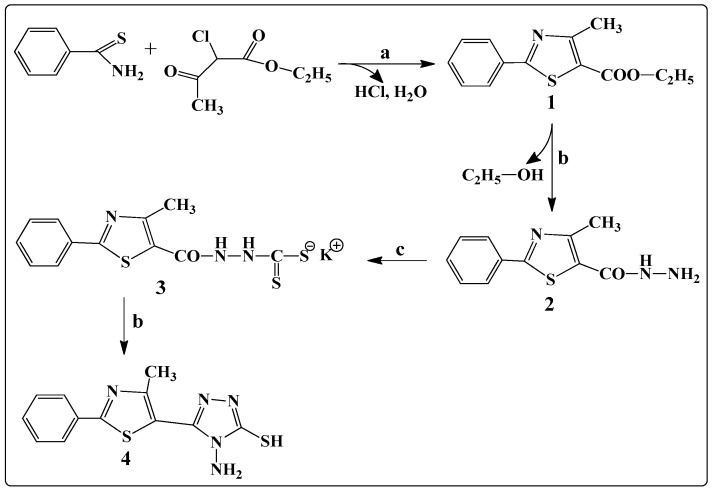
The synthesis of 4-amino-5-(4-methyl-2-phenylthiazol-5-yl)-4*H*-1,2,4-triazole-3-thiol. ^a^ EtOH, reflux; ^b^ H_2_N-NH_2_/EtOH, reflux; ^c^ CS_2_/KOH, room temperature.

**Scheme 2:**
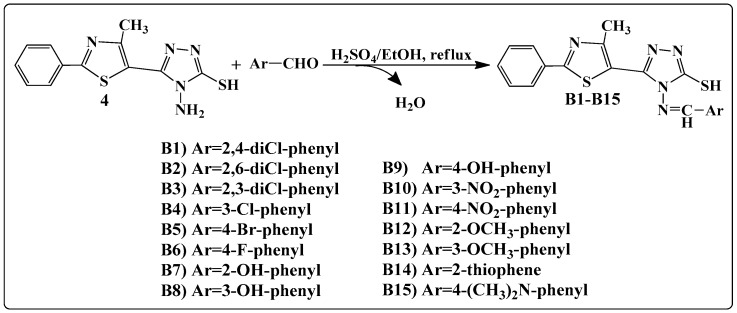
Synthesis of Schiff bases.

The general synthetic route for the synthesis of 4-amino-5-(4-methyl-2-phenylthiazol-5-yl)-4*H*-1,2,4-triazole-3-thiol is illustrated in Scheme 1. The general method known as Hantzsch condensation has been used for the synthesis in good yields (85%–90%) of 4-methyl-2-phenylthiazole carboxylate **1**. The 4-methyl-2-phenylthiazole-5-carbohydrazide **2** was obtained by treating compound **1** with hydrazine hydrate, under reflux, in a water bath, for 3 h. Then, compound **2** was treated with CS_2_ and KOH, at room temperature, to give the potassium 2-(4-methyl-2-phenylthiazole-5-carbonyl)hydrazine-carbodithioate **3**. Finally, the 4-amino-5-(4-methyl-2-phenylthiazol-5-yl)-4*H*-1,2,4-triazole-3-thiol **4** was obtained, in good yields (80%), by treating compound **3** with hydrazine hydrate, under reflux, for 2 h. Schiff bases **B1**–**B15** were synthesized in good yields by the condensation of compound **4** with various aromatic or heteroaromatic aldehydes, in the presence of concentrated H_2_SO_4_, used as a catalyst (Scheme 2).

The structures of thiazolyl-triazole Schiff bases were established by elemental analysis and on the basis of their mass spectra (MS), infrared spectra (IR), nuclear magnetic resonance (^1^H-NMR and ^13^C-NMR) spectra. The results of the C, H, N, S quantitative elemental analysis of all synthesized compounds were in accordance with the calculated values, within ±0.4% of the theoretical values. The spectral data confirmed the formation of the **B1**–**B15** Schiff bases. Details of the synthetic procedures, the yields and the physical, analytical and spectral data of the synthesized compounds are presented in the Experimental Section. Compounds **1**–**3** were previously reported in the literature [13,14].

Mass spectra recorded for the final products **B1**–**B15** gave an idea about the fragmentation of the compounds with their corresponding mass and, in all cases, revealed the correct molecular ion peaks (M + 1), as suggested by their molecular formulas. The absence of the NH_2_ asymmetric and symmetric stretching vibrations at 3281 cm^−1^ and 3186 cm^−1^ and also the presence of N=CH stretch absorption bands at 1635–1618 cm^−1^ in the final compounds’ IR spectra provided strong evidences for the formation of Schiff bases **B1**–**B15**.

**Figure 1:**
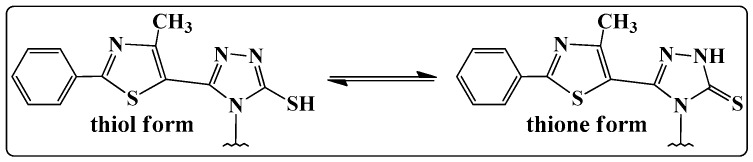
Thiol-thione tautomerism shown by the synthesized Schiff bases.

In the ^1^H-NMR spectrum of compound **4**, a signal was recorded, characteristic for the amino protons, as a singlet, at 5.73 ppm. The absence of this signal from the ^1^H-NMR spectra of the new synthesized compounds (**B1**–**B15**), and also the presence of a singlet characteristic to the N=CH proton at 9.36–9.52 ppm further confirmed the condensation between the 4-amino-5-(4-methyl-2-phenylthiazol-5-yl)-4*H*-1,2,4-triazole-3-thiol **4** and the corresponding aromatic aldehydes. The 3-mercapto-1,2,4-triazoles could exist in two tautomeric forms (Figure 1), because the labile hydrogen may be attached either to the nitrogen (the thione form) or to the sulphur atom (the thiol form) [15].

In the IR spectra of compounds **4** and **B1**–**B15,** absorption peaks were observed at 3131–3086 cm^−1^; due to the stretching vibration of the N-H group from the triazole ring, the absorption bands were at 1288–1257 cm^−1^, corresponding to C=S stretching vibrations and the absence of the thiol SH absorption band at about 2600–2550 cm^−1^ (characteristic for the thiol form), proving that the compounds were in thione form, in solid state. Furthermore, in the 13.91–14.18 ppm region of the ^1^H-NMR spectra of compounds **4** and **B1**–**B15**, the NH proton resonated as a singlet, indicating that the crystal structures of the compounds correspond to the thione form. The ^13^C-NMR spectra of the newly synthesized compounds were consistent with the proposed structures and exhibited only the presence of thionic tautomeric form (C=S) through the presence of the exocyclic C=S peaks, at 169.88–170.25 ppm. The spectral investigations performed by us proved that thiazolyl-triazole Schiff bases **B1**–**B15** exist in their thione form, in solid state.

### 2.2. Antifungal Activity

#### 2.2.1. Determination of Inhibition Zone Diameters

The anti-*Candida* activity was tested in vitro using the disk diffusion method, by measuring the diameters of the inhibition zones. The synthesized compounds **B1**–**B15** were screened against the fungal strain of *Candida albicans* ATCC 90028. The concentrations used for evaluating the antimicrobial activity were 100 μg/disk for the synthesized compounds and for the reference substance used, fluconazole. Because the compounds are not soluble in water, the solvent used to prepare the solutions of the synthesized compounds, dimethylsulfoxide (DMSO), exhibited no inhibitory activity on the fungal strain used in the study. The obtained results are presented in Table 1, compared to fluconazole.

Analyzing the results obtained, we can observe that all the tested compounds showed zone inhibition diameters inferior to Fluconazole, used as an antifungal reference drug. Concerning the relationships between the structure of the compounds and the anti-*Candida* activity, we observed that the compounds substituted on the phenyl bound to the azomethinic group with *para*-Br (**B5**), *meta*-nitro (**B10**) and *para*-nitro (**B11**) showed the biggest zone inhibition diameters (20 mm). The other compounds, with the exception of the 2,4-dichloro substituted compound **B1** (16 mm), showed the same diameter (18 mm).

#### 2.2.2. Determination of Minimum Inhibitory Concentration (MIC) and Minimum Fungicidal Concentration (MFC) Values

In the first screening test, we observed that the compounds showed similar anti-*Candida* activity, eleven Schiff bases having the same inhibition zone diameters (Table 1). In order to analyze the relationships between the structure of the **B1**–**B15** compounds and their anti-*Candida* activity, we used two less virulent *Candida albicans* strains (*C. albicans* ATCC 10231 and *C. albicans* ATCC 18804) and a non-albicans *Candida* strain (*Candida krusei*) [16,17,18], for the minimum inhibitory concentration (MIC) and for the minimum fungicidal concentration (MFC) tests.

The broth microdilution method was employed for the MIC test. All the synthesized compounds were tested against three strains of fungi (*Candida albicans* ATCC 10231, *Candida albicans* ATCC 18804 and *Candida krusei* ATCC 6258). Stock solutions (1 mg/mL) were prepared by dissolving the test compounds and the reference antimicrobials (fluconazole, ketoconazole) in sterile DMSO. The results are presented in Table 2 and Table 3.

From the obtained results, we can observe that all the tested compounds showed MIC values equal or inferior to systemic antifungal reference drug, Fluconazole (Table 2). The Schiff base **B10** (*meta*-nitro) is 4-fold more active than Fluconazole and 2-fold more active than the other reference local antifungal drug, Ketoconazole, respectively, on *Candida albicans* ATCC 10231. On *C. albicans* ATCC 18804, compounds **B8** (*meta*-hydroxy), **B10** and **B14** (substituted with thiophene heterocycle) were 2-fold more active than Fluconazole and equal, as potential, with Ketoconazole. Concerning the non-albicans *Candida* strain, compounds **B5** (*para*-Br) and **B10** were 2-fold more active than Fluconazole and with the same activity as Ketoconazole.

Determination of MFC confirmed the results previously obtained when MIC was investigated. Supplementary, we observed that Schiff bases B8 (*meta*-hydroxy), B9 (*para*-hydroxy) and B10 presented the same activity against *C. albicans* ATCC 18804. 

Finally, we remarked that the thiazolyl-triazole Schiff base **B10** can be considered the most promising anti-*Candida* candidate, the compound being more active than Fluconazole and with similar activity as Ketoconazole, on the three tested *Candida* strains. The MFC/MIC ratio for all tested compounds was 2, suggesting that the synthesized Schiff bases could act as fungicidal agents [19].

### 2.3. Molecular Docking

With the aim of elucidating the mechanism of action of synthesized Schiff bases, molecular docking studies were performed on lanosterol-14α-demethylase from *Saccharomyces cerevisiae*. To check the settings and the accuracy of the docking method, we performed a re-docking of itraconazole, the former co-crystallized ligand with CYP51 in its active site. The root mean square deviation (RMSD) value between the top ranked predicted conformation and the observed X-ray crystal structure of the Protein Data Bank (PDB) deposited structure, was 0.324 Å. A value being below 2 Å the docking protocol was validated.

The synthesized compounds **B1**–**B15** and fluconazole, as a control inhibitor, were docked into the active site of lanosterol 14α-demethylase. The predicted binding affinity of Schiff bases into the active site of the enzyme and the predicted polar contacts between them, are presented in Table 4.

**Figure 2:**
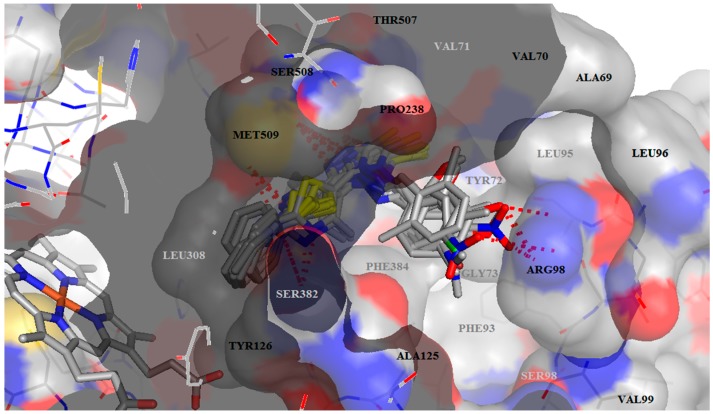
Docking results—A general view with all ligands inserted in the active site of CYP51. The active site and the ligands are depicted as sticks. The hydrogen bonds are depicted as dashed red lines. The foreground amino acids are in grey.

Our studies showed that all of our thiazolyl-triazoles Schiff bases **B1**–**B15** did not covalently interact with the heme from the active site of lanosterol 14α-demethylase, like classic antifungal azoles. They interact with the amino acids in the access channel to the active site of the lanosterol 14α-demethylase (Figure 2, Table 4). All the compounds form hydrogen bonds between the thiazole nitrogen (N1) with Met509 or Ser382 and between the triazole nitrogens (N2, N3) with the residues Phe506 and Ser508. We also observed that the Schiff bases carrying a hydroxy-phenol substituent in *meta* position (**B8**) and the corresponding *meta-* and *para*-substituted nitro-derivatives (**B10**, **B11**) form supplementary bonds with the Arg98 from the access groove (Figure 2).

Despite the role of the azole as an important pharmacophore for the antimycotic activity, it represents also a key toxicophore for the hepatotoxicity of azole antifungal drugs, due to the coordination binding of its nitrogen atom to the iron atom of heme [20]. Because the affinity of our Shiff bases for CYP51 was attributed to the non-covalent interaction with the aminoacids from the access channel to the active site, the studies presented here can be exploited to develop new antifungal agents that specifically interact with the residues from the active site, avoiding the serious toxicity of classic azoles, which arises from coordination binding with the heme of mammalian CYTP450s.

**Figure 3:**
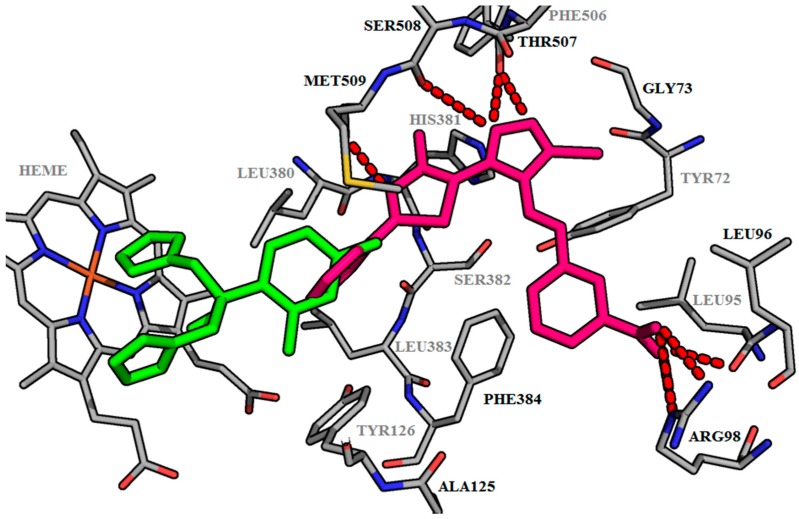
Binding mode of Fluconazole and **B10**. The active site of lanosterol 14α-demethylase is depicted as sticks. The Fluconazole is depicted in green; the Schiff base **B10** in pink. The hydrogen bonds are depicted as dashed red lines. The foreground amino acids are in grey.

The data obtained from docking shows that all the Schiff bases have higher binding interaction energy, ranging from −10.45 to −12.92 kcal/mol relative to fluconazole (−7.03 kcal/mol) (Table 4). Figure 3 shows the representation of the binding patterns into the active site of CYP51 for the fluconazole (green), used as the reference antifungal drug and for the Schiff base **B10** (pink) which showed the highest inhibition capacity. Non-interacting amino acids in the foreground were removed for clarity. The thiole group is not supposed to interact with amino acids from the enzyme. Our future studies will be directed towards optimizing the structure, in order to obtain compounds with a superior binding affinity and with better anti-*Candida* activity. Modulation of thiole by introducing one polar substituent, may improve the inhibition ability, because of the possible supplementary interaction with Tyr72.

### 2.4. ADME and Molecular Property Prediction

High oral bioavailability is an important factor for the development of bioactive compounds as therapeutic agents [21]. The predictors for this are represented by a good intestinal absorption, a reduced molecular flexibility, low polar surface area and hydrogen-bounding capacity [22]. All our compounds pass Lipinski’s “Rule of 5” (Table 5). Number of rotatable bonds is important for conformational changes of molecules and consequently, for the binding of receptors or channels. All tested compounds pass one of the oral bioavailability criteria, having less than 10 rotatable bonds and no chirality center, therefore exhibiting low conformational flexibility. All compounds have molar refractivity under 130.

Topological polar surface area (tPSA), that is the surface belonging to polar atoms, is a descriptor that correlates well with passive molecular transport through membranes, including the blood–brain barrier [23]. Compounds **B1**–**6**, **B12**, **B13** and **B15** have tPSA values less than 140 Å^2^, passing the criteria for gastro-intestinal absorption, after oral administration (Table 5). The other compounds—**B7**–**11** with hydroxyl, nitro groups and thiophene substituted compound **B14**—violate this rule. The absorption percent of all the Schiff bases ranges from 50% to 66%, which is an indication of an acceptable bioavailability by oral route (>50%). On the other hand, all the compounds were predicted to have a low blood–brain barrier penetration (tPSA > 90 Å^2^), so, the risks of central nervous system (CNS) side effects are reduced or absent. Water solubility of our compounds is poor, for all compounds—logS value being between −6 and −10. The compounds **B1**–**B5** are considered “poorly soluble” and the rest are “moderately soluble”.

All Schiff bases are predicted as noninhibitors of CYP2D6, therefore the side effects (i.e., liver dysfunction) are not expected upon the administration of these compounds. Also, all are predicted to be metabolized by CYP2C9 [24,25], due to the presence of the acidic thiol group.

P-glycoprotein (P-gp) is a member of the ATP-binding cassette (ABC) transporter family involved in intestinal absorption, drug metabolism, and brain penetration, and its inhibition can seriously alter a drug’s bioavailability and safety [26]. The drug induced phospholipidosis is a disorder characterized by the excess accumulation of phospholipids in tissues and is associated with drug induced toxicity [27]. The results obtained showed that none of our Schiff bases is a substrate for P-gp and none induce phospholipidosis.

According to the above ADME and toxicity results, the thiazolyl-triazole Schiff bases **B1**–**15** show good pharmacokinetic properties, but with some limitations, such as moderate gastrointestinal absorption and no blood–brain barrier penetration. All molecules are accepted as drug-like, passing Lipinski’s “Rule of five”. The predicted parameters are within the range of accepted values. However, further optimization should be performed on tested compounds, in order to achieve better ADME properties. A mandatory improvement for the properties of the newly synthesized derivatives would be to increase their bioavailability. Small chemical modulations on the molecules could improve the ADME properties. Potential chemical modulations, such as alkylation, on the ionisable thiol moiety, would severely change the ADME properties of the resulted molecules, in order to extend the potential oral administration.

## 3. Materials and Methods

### 3.1. Chemistry

All chemicals and reagents were obtained from commercial sources and were used as supplied, without further purification. Compounds **1**–**3** were previously reported and were synthesized by us according to methodologies described in the literature [13,14].

Melting points were determined with an Electrothermal melting point meter in the open glass capillary method and are uncorrected. The reaction progress and purity of the synthesized compounds were monitored by analytical thin layer chromatography (TLC) using Merck precoated Silica Gel 60F_254_ sheets (Darmstadt, Germany), heptane–ethyl-acetate 3:7 elution system and ultraviolet UV light (254 nm) for visualization. MS analyses were performed at 70 eV with an Agilent gas chromatograph 6890 (Darmstadt, Germany) equipped with an apolar Macherey Nagel Permabond SE 52 capillary column (Dueren, Germany) and with an LCMS Shimadzu Mass Spectrometer (Shimadzu Corporation, Columbia, MD, USA). Elemental analysis was registered with a Vario El CHNS instrument (Hanau, Germany). IR spectra were recorded on a JASCO FT-IR-4100 spectrometer (Cremella, Italy) using the ATR technique (Attenuated Total Reflectance). Nuclear magnetic resonance (^1^H-NMR) spectra were recorded at room temperature on a Bruker Avance NMR spectrometer (Karlsruhe, Germany) operating at 400 and 500 MHz, using tetramethylsilane (TMS) as an internal standard (chemical shift in *δ* ppm) and were in accordance with the assigned structures. The samples were prepared by dissolving the compounds in DMSO-*d*_6_ (*δ*_H_ = 2.51 ppm) as a solvent and spectra were recorded using a single excitation pulse of 12 μs (^1^H-NMR). Spin multiplets are given as *s* (singlet), *d* (doublet), *t* (triplet) and *m* (multiplet). ^13^C-NMR spectra were recorded on a Bruker Avance NMR spectrometer (Karlsruhe, Germany), operating at 125 MHz, in DMSO-*d*_6_, using a waltz-16 decoupling scheme.

*General procedure for the synthesis of ethyl 4-methyl-2-phenylthiazole-5-carboxylate* (**1**) [13]. An amount of 1 mmol (0.137 g) of thiobenzamide was dissolved in 10 mL of ethanol and 0.20 mL of ethyl 2-chloroacetoacetate was added. The reaction mixture was refluxed for 3 h on a water bath. After cooling, the reaction mixture was poured onto a mixture of ice and water. The formed precipitate was filtered, then washed with distilled water, to remove traces of the remaining halocarbonyl and then vacuum dried.

*General procedure for the synthesis of 4-methyl-2-phenylthiazole-5-carbohydrazide* (**2**) [14]. An amount of 117.4 mmol (5.87 g) of hydrazine hydrate was added to the alcoholic solution of the carboxylate of 4-methyl-2-phenylthiazole (**1**) (58.7 mmol in 100 mL absolute ethanol). The resulting reaction mixture was refluxed on a water bath, at 100 °C, for 4 h, and then it was concentrated under reduced pressure. Distilled water was added to the obtained precipitate and then the mixture was subjected to low pressure filtration, washed with distilled water, dried and recrystallized from absolute ethanol.

*General procedure for the synthesis of potassium 2-(4-methyl-2-phenylthiazole-5-carbonyl)hydrazinecarbodithioate* (**3**) [14]. To the alcoholic solution of 4-methyl-2-phenylthiazol-5-carbohydrazide **2** (20 mmol in 40 mL of alcohol) was added a suspension of 30 mmol (1.68 g) of KOH in 60 mL of absolute ethanol and 30 mmol (3.04 g) CS_2_. The reaction mixture was stirred for 3 h at room temperature to obtain a quantitative precipitation. The reaction mass was filtered, washed with diethyl ether and dried in order to yield a bright yellow precipitate.

*General procedure for the synthesis of 4-amino-5-(4-methyl-2-phenylthiazol-5-yl)-4H-1,2,4-triazole-3-thiol* (**4**). The alcoholic solution of potassium 2-(4-methyl-2-phenylthiazole-5-carbonyl) hydrazinecarbodithioate **3** (10 mmol in 40 mL of absolute ethanol) was treated with 10 mmol of hydrazine hydrate (80%). The reaction mixture was refluxed for 2 h. After cooling, the reaction mass was poured onto water and then acidified with a HCl (10%) solution. The reaction mixture was allowed to stand for 2 h for quantitative precipitation and evolution of H_2_S. The obtained precipitate was filtered and washed with ethanol. The obtained compound was recrystallized from absolute ethanol. Yield 80.0% (0.23 g); m.p. 165 °C; light yellow powder; Anal. Calcd for C_12_H_11_N_5_S_2_ (289.38): C, 49.81; H, 3.83; N, 24.20; S, 22.16; Found: C, 49.83; H, 3.85; N, 24.24; S, 22.13; IR (ATR, cm^−1^): 3281 (ν NH_2_ asym), 3186 (ν NH_2_ sym), 3110 (ν NH_triazole_), 1268 (ν C=S); ^1^H-NMR (500 MHz, DMSO-*d_6_*, *δ*/ppm): 14.09 (s, 1H, NH), 7.92 (d, 2H, ArH), 7.68 (m, 2H, ArH), 7.59 (m, 1H, ArH), 5.73 (s, 2H, NH_2_), 2.54 (s, 3H, CH_3_); ^13^C-NMR (125 MHz, DMSO-*d*_6_, *δ*/ppm): 170.16 (C=S), 159.34 (C), 153.51 (C), 151.07 (C), 144.12 (C), 143.81 (C), 131.12 (2CH), 129.41 (2CH), 127.85 (CH), 16.05 (CH_3_); MS (EI, 70 eV) *m*/*z* (%): 290.08 (M + 1).

*General procedure for the synthesis of* *Schiff bases* **B1**–**B15**. An amount of 2 mmol (0.578 g) of 4-amino-5-(4-methyl-2-phenylthiazol-5-yl)-4*H*-1,2,4-triazole-3-thiol **4** was suspended in 10 mL of absolute ethanol. To the resulting suspension was added an alcoholic solution of 2 mmol of aldehyde (aromatic or heteroaromatic) in 5 mL of absolute ethanol and 2–3 drops of concentrated H_2_SO_4_ as a catalyst. The reaction mixture was refluxed for 6 h. The obtained precipitate was filtered hot and washed with absolute ethanol, and then it was dried and recrystallized from DMSO.

*4-(2,4-Dichlorobenzylideneamino)-5-(4-methyl-2-phenylthiazol-5-yl)-4H-1,2,4-triazole-3-thiol* (**B1**). Yield 67.4% (0.300 g); m.p. 247–249 °C; light yellow powder; Anal. Calcd for C_19_H_13_Cl_2_N_5_S_2_ (446.38): C, 51.12; H, 2.31; N, 15.68; S, 14.33; Found: C, 51.32; H, 2.32; N, 15.61; S, 14.36; IR (ATR, cm^−1^): 3095 (ν NH_triazole_), 1633 (ν -N=CH-), 1260 (ν C=S); 1165 (ν C-Cl), 1098 (ν C-Cl); ^1^H-NMR (500 MHz, DMSO-*d_6_*, *δ*/ppm): 14.15 (s, 1H, NH), 9.42 (s, 1H, -N=CH-), 7.93–8.01 (d, 3H, ArH), 7.72 (s, 1H, ArH), 7.50 (d, 1H, ArH), 7.35–7.42 (m, 3H, ArH), 2.33 (s, 3H, CH_3_); ^13^C-NMR (125 MHz, DMSO-*d*_6_, *δ*/ppm): 170.25 (C=S), 159.14 (C), 157.73 (CH=N), 154.01 (C), 150.98 (C), 144.11 (C), 132.44 (C), 131.09 (C), 130.89 (2CH), 129.33 (2CH), 129.15 (CH), 129.03 (CH), 128.82 (CH), 128.41 (2C), 126.01 (CH), 15.93 (CH_3_); MS (EI, 70 eV) *m*/*z* (%): 446.3 (M + 1).

*4-(2,6-Dichlorobenzylideneamino)-5-(4-methyl-2-phenylthiazol-5-yl)-4H-1,2,4-triazole-3-thiol* (**B2**). Yield 70.5% (0.314 g); m.p. 300 °C; light yellow powder; Anal. Calcd for C_19_H_13_Cl_2_N_5_S_2_ (446.38): C, 51.12; H, 2.31; N, 15.68; S, 14.33; Found: C, 50.9; H, 2.32; N, 15.61; S, 14.27; IR (ATR, cm^−1^): 3092 (ν NH_triazole_), 1635 (ν -N=CH-), 1265 (ν C=S); 1161 (ν C-Cl), 1095 (ν C-Cl); ^1^H-NMR (500 MHz, DMSO-*d_6_*, *δ*/ppm): 14.11 (s, 1H, NH), 9.41 (s, 1H, -N=CH-), 7.95–8.03 (d, 2H, ArH), 7.54–7.59 (m, 4H, ArH), 2.39 (s, 3H, CH_3_); ^13^C-NMR (125 MHz, DMSO-*d*_6_, *δ*/ppm): 170.09 (C=S), 159.16 (C), 157.61 (CH=N), 153.97 (C), 151.04 (C), 144.01 (C), 133.72 (2C), 132.19 (C), 130.96 (2CH), 129.37 (2CH), 129.11 (CH), 128.75 (CH), 128.64 (C), 128.32 (2CH), 15.86 (CH_3_); MS (EI, 70 eV) *m*/*z* (%): 446.3 (M + 1).

*4-(2,3-Dichlorobenzylideneamino)-5-(4-methyl-2-phenylthiazol-5-yl)-4H-1,2,4-triazole-3-thiol* (**B3**). Yield 77.1% (0.687 g); m.p. 285–290 °C; light yellow powder; Anal. Calcd for C_19_H_13_Cl_2_N_5_S_2_ (446.38): C, 51.12; H, 2.31; N, 15.68; S, 14.33; Found: C, 51.32; H, 2.32; N, 15.61; S, 14.27; IR (ATR, cm^−1^): 3096 (ν NH_triazole_), 1632 (ν -N=CH-), 1263 (ν C=S); 1159 (ν C-Cl), 1091 (ν C-Cl); ^1^H-NMR (500 MHz, DMSO-*d*_6_, *δ*/ppm): 14.06 (s, 1H, NH), 9.48 (s, 1H, -N=CH-), 7.89–8.93 (d, 2H, ArH), 7.71 (d, 1H, ArH), 7.54 (d, 1H, ArH), 7.35-7.48 (m, 4H, ArH), 2.36 (s, 3H, CH_3_); ^13^C-NMR (125 MHz, DMSO-*d*_6_, *δ*/ppm): 170.04 (C=S), 159.11 (C), 157.75 (CH=N), 153.97 (C), 151.04 (C), 144.01 (C), 142.88 (C), 134.65 (C), 132.19 (CH), 130.98 (2CH), 130.44 (C), 129.33 (2CH), 128.88 (CH), 128.61 (C), 128.33 (CH), 125.12 (CH), 15.79 (CH_3_); MS (EI, 70 eV) *m*/*z* (%): 446.3 (M + 1).

*4-(3-Chlorobenzylideneamino)-5-(4-methyl-2-phenylthiazol-5-yl)-4H-1,2,4-triazole-3-thiol* (**B4**). Yield 82.92% (0.781 g); m.p. 275 °C; light yellow powder; Anal. Calcd for C_19_H_14_ClN_5_S_2_ (411.9): C, 55.34; H, 3.39; N, 16.99; S, 15.53; Found: C, 55.6; H, 3.37; N, 17.05; S, 15.59; IR (ATR, cm^−1^): 3091 (ν NH_triazole_), 1630 (ν -N=CH-), 1271 (ν C=S); 1132 (ν C-Cl); ^1^H-NMR (500 MHz, DMSO-*d*_6_, *δ*/ppm): 14.18 (s, 1H, NH), 9.52 (s, 1H, -N=CH-), 7.97–8.06 (d, 2H, ArH), 7.92 (s, 1H, ArH), 7.77 (d, 1H, ArH), 7.59 (d, 1H, ArH), 7.47–7.54 (m, 4H, ArH), 2.41 (s, 3H, CH_3_); ^13^C-NMR (125 MHz, DMSO-*d*_6_, *δ*/ppm): 170.12 (C=S), 159.15 (C), 157.66 (CH=N), 153.81 (C), 151.07 (C), 143.96 (C), 135.16 (C), 134.51 (C), 131.21 (CH), 130.93 (2CH), 130.29 (CH), 129.29 (2CH), 128.94 (CH), 128.68 (C), 127.36 (CH), 127.14 (CH), 15.92 (CH_3_); MS (EI, 70 eV) *m*/*z* (%): 412.04 (M + 1).

*4-(4-Bromobenzylideneamino)-5-(4-methyl-2-phenylthiazol-5-yl)-4H-1,2,4-triazole-3-thiol* (**B5**). Yield 75.3% (0.343 g); m.p. 296–298 °C; yellow powder; Anal. Calcd for C_19_H_14_BrN_5_S_2_ (456.38): C, 49.89; H, 3.06; N, 15.33; S, 14.02; Found: C, 50.1; H, 3.07; N, 15.33; S, 14.07; IR (ATR, cm^−1^): 3104 (ν NH_triazole_), 1618 (ν -N=CH-), 1274 (ν C=S); 1055 (ν C-Br); ^1^H-NMR (500 MHz, DMSO-*d*_6_, *δ*/ppm): 14.12 (s, 1H, NH), 9.40 (s, 1H, -N=CH-), 7.98–8.04 (d, 2H, ArH), 7.81–7.83 (d, 2H, ArH), 7.60 (d, 2H, ArH), 7.44–7.49 (m, 3H, ArH), 2.35 (s, 3H, CH_3_); ^13^C-NMR (125 MHz, DMSO-*d*_6_, *δ*/ppm): 170.02 (C=S), 159.16 (C), 157.64 (CH=N), 153.61 (C), 151.17 (C), 143.67 (C), 131.85 (2CH), 131.11 (C), 130.89 (2CH), 129.29 (2CH), 128.93 (CH), 128.71 (C), 128.30 (2CH), 125.59 (C), 15.77 (CH_3_); MS (EI, 70 eV) *m*/*z* (%): 456.3 (M + 1).

*4-(4-Fluorobenzylideneamino)-5-(4-methyl-2-phenylthiazol-5-yl)-4H-1,2,4-triazole-3-thiol* (**B6**). Yield 67.8% (0.268 g); m.p. 294–296 °C; yellow powder; Anal. Calcd for C_19_H_14_FN_5_S_2_ (395.48): C, 57.65; H, 3.54; N, 17.7; S, 16.18; Found: C, 57.9; H, 3.55; N, 17.62 S, 16.11; IR (ATR, cm^−1^): 3107 (ν NH_triazole_), 1620 (ν -N=CH-), 1270 (ν C=S); 1215 (ν C-F); ^1^H-NMR (500 MHz, DMSO-*d*_6_, *δ*/ppm): 13.99 (s, 1H, NH), 9.47 (s, 1H, -N=CH-), 7.96–8.01 (d, 2H, ArH), 7.79–7.82 (d, 2H, ArH), 7.46–7.54 (m, 3H, ArH), 7.31 (d, 2H, ArH), 2.29 (s, 3H, CH_3_); ^13^C-NMR (125 MHz, DMSO-*d*_6_, *δ*/ppm): 169.96 (C=S), 165.22 (C), 159.20 (C), 157.58 (CH=N), 153.65 (C), 151.18 (C), 143.53 (C), 130.98 (2CH), 130.81 (2CH), 129.33 (2CH), 128.95 (CH), 128.68 (C), 127.77 (C), 115.59 (2CH), 15.83 (CH_3_); MS (EI, 70 eV) *m*/*z* (%): 396.3 (M + 1).

*2-((3-Mercapto-5-(4-methyl-2-phenylthiazol-5-yl)-4H-1,2,4-triazol-4-ylimino) methyl)phenol* (**B7**). Yield 46.8% (0.356 g); m.p. 269 °C; yellow powder; Anal. Calcd for C_19_H_15_N_5_OS_2_ (393.49): C, 57.94; H, 3.81; N, 17.78; S, 16.26; Found: C, 58.2; H, 3.82; N, 17.85 S, 16.19; IR (ATR, cm^−1^): 3348 (ν O-H), 3115 (ν NH_triazole_), 1626 (ν -N=CH-), 1284 (ν C=S); 1243 (ν C-O); ^1^H-NMR (500 MHz, DMSO-*d*_6_, *δ*/ppm): 13.97 (s, 1H, NH), 9.82 (s, 1H, OH), 9.43 (s, 1H, -N=CH-), 7.99–8.05 (d, 2H, ArH), 7.68 (d, 1H, ArH), 7.44–7.46 (m, 4H, ArH), 7.15 (m, 1H, ArH), 7.01 (d, 1H, ArH), 2.27 (s, 3H, CH_3_); ^13^C-NMR (125 MHz, DMSO-*d*_6_, *δ*/ppm): 169.99 (C=S), 161.31 (C), 159.23 (C), 157.88 (CH=N), 153.57 (C), 151.21 (C), 143.51 (C), 132.53 (CH), 132.15 (CH), 130.85 (2CH), 129.41 (2CH), 128.90 (CH), 128.59 (C), 121.76 (CH), 118.18 (C), 116.99 (CH), 15.88 (CH_3_); MS (EI, 70 eV) *m*/*z* (%): 394.2 (M + 1).

*3-((3-Mercapto-5-(4-methyl-2-phenylthiazol-5-yl)-4H-1,2,4-triazol-4-ylimino) methyl)phenol* (**B8**). Yield 65.3% (0.496 g); m.p. 295–297 °C; light yellow powder; Anal. Calcd for C_19_H_15_N_5_OS_2_ (393.49): C, 57.94; H, 3.81; N, 17.78; S, 16.26; Found: C, 58.2; H, 3.82; N, 17.85 S, 16.19IR (ATR, cm^−1^): 3344 (ν O-H), 3112 (ν NH_triazole_), 1622 (ν -N=CH-), 1281 (ν C=S); 1245 (ν C-O); ^1^H-NMR (500 MHz, DMSO-*d*_6_, *δ*/ppm): 13.92 (s, 1H, NH), 9.78 (s, 1H, OH), 9.39 (s, 1H, -N=CH-), 8.01–8.04 (d, 2H, ArH), 7.54 (s, 1H, ArH), 7.48–7.56 (m, 3H, ArH), 7.39 (d, 1H, ArH), 7.23 (m, 1H, ArH), 6.98 (d, 1H, ArH), 2.34 (s, 3H, CH_3_); ^13^C-NMR (125 MHz, DMSO-*d_6_*, *δ*/ppm): 169.92 (C=S), 159.09 (C), 158.83 (C), 157.67 (CH=N), 153.46 (C), 151.24 (C), 143.44 (C), 135.33 (C), 130.87 (2CH), 130.27 (CH), 129.30 (2CH), 128.92 (CH), 128.64 (C), 121.74 (CH), 118.26 (CH), 115.01 (CH), 15.93 (CH_3_); MS (EI, 70 eV) *m*/*z* (%): 394.2 (M + 1).

*4-((3-Mercapto-5-(4-methyl-2-phenylthiazol-5-yl)-4H-1,2,4-triazol-4-ylimino) methyl)phenol* (**B9**). Yield 68.15% (0.518 g); m.p. 285 °C; light yellow powder; Anal. Calcd for C_19_H_15_N_5_OS_2_ (393.49): C, 57.94; H, 3.81; N, 17.78; S, 16.26; Found: C, 57.7; H, 3.82; N, 17.7 S, 16.19; IR (ATR, cm^−1^): 3341 (ν O-H), 3118 (ν NH_triazole_), 1623 (ν -N=CH-), 1285 (ν C=S); 1249 (ν C-O); ^1^H-NMR (500 MHz, DMSO-*d*_6_, *δ*/ppm): 14.07 (s, 1H, NH), 9.83 (s, 1H, OH), 9.38 (s, 1H, -N=CH-), 7.78–7.95 (d, 4H, ArH), 7.41–7.47 (m, 3H, ArH), 6.98 (d, 2H, ArH), 2.32 (s, 3H, CH_3_); ^13^C-NMR (125 MHz, DMSO-*d_6_*, *δ*/ppm): 169.94 (C=S), 160.39 (C), 158.88 (2C), 158.42 (C), 157.97 (CH=N), 143.74 (C), 130.97 (2CH), 130.52 (2CH), 129.36 (2CH), 128.94 (CH), 126.33 (C), 116.11 (2CH), 106.06 (C), 16.24 (CH_3_); MS (EI, 70 eV) *m*/*z* (%): 394.2 (M + 1).

*5-(4-Methyl-2-phenylthiazol-5-yl)-4-(3-nitrobenzylideneamino)-4H-1,2,4-triazole-3-thiol* (**B10**)*.* Yield 80.8% (0.682 g); m.p. 270 °C; light yellow powder; Anal. Calcd for C_19_H_14_N_6_O_2_S_2_ (422.48): C, 53.96; H, 3.31; N, 19.88; S, 15.14; Found: C, 53.7; H, 3.32; N, 19.8; S, 15.2; IR (ATR, cm^−1^): 3131 (ν NH_triazole_), 1521 (ν N-O asy), 1629 (ν -N=CH-), 1324 (ν N-O sy), 1257 (ν C=S); ^1^H-NMR (500 MHz, DMSO-*d*_6_, *δ*/ppm): 14.04 (s, 1H, NH), 9.34 (s, 1H, -N=CH-), 8.52 (s, 1H, ArH), 8.01–8.22 (d, 4H, ArH), 7.49–7.59 (m, 4H, ArH), 2.45 (s, 3H, CH_3_); ^13^C-NMR (125 MHz, DMSO-*d*_6_, *δ*/ppm): 169.88 (C=S), 159.03 (C), 157.76 (CH=N), 153.45 (C), 151.19 (C), 148.88 (C), 143.66 (C), 135.44 (CH), 134.58 (C), 130.83 (2CH), 129.71 (CH), 129.38 (2CH), 128.83 (CH), 128.55 (C), 126.36 (CH), 121.65 (CH), 16.12 (CH_3_); MS (EI, 70 eV) *m*/*z* (%): 423.2 (M + 1).

*5-(4-Methyl-2-phenylthiazol-5-yl)-4-(4-nitrobenzylideneamino)-4H-1,2,4-triazole-3-thiol* (**B11**)*.* Yield 50% (0.412 g); m.p. 275 °C; orange powder; Anal. Calcd for C_19_H_14_N_6_O_2_S_2_ (422.48): C, 53.96; H, 3.31; N, 19.88; S, 15.14; Found: C, 53.7; H, 3.29; N, 19.95; S, 15.2; IR (ATR, cm^−1^): 3129 (ν NH_triazole_), 1525 (ν N-O asym), 1625 (ν -N=CH-), 1320 (ν N-O sym), 1259 (ν C=S); ^1^H-NMR (500 MHz, DMSO-*d*_6_, *δ*/ppm); 13.93 (s, 1H, NH), 9.36 (s, 1H, -N=CH-), 8.02–8.29 (d, 6H, ArH), 7.45–7.51 (m, 3H, ArH), 2.34 (s, 3H, CH_3_); ^13^C-NMR (125 MHz, DMSO-*d*_6_, *δ*/ppm): 169.91 (C=S), 159.09 (C), 157.78 (CH=N), 153.35 (C), 151.16 (C), 150.13 (C), 143.59 (C), 138.54 (C), 130.91 (2CH), 129.25 (2CH), 128.90 (CH), 128.57 (C), 127.89 (2CH), 124.16 (2CH), 16.16 (CH_3_); MS (EI, 70 eV) *m*/*z* (%): 423.2 (M + 1).

*4-(2-Methoxybenzylideneamino)-5-(4-methyl-2-phenylthiazol-5-yl)-4H-1,2,4-triazole-3-thiol* (**B12**). Yield 71.7% (0.603 g); m.p. 285 °C; yellow powder; Anal. Calcd for C_20_H_17_N_5_OS_2_ (407.51): C, 58.89; H, 4.17; N, 17.17; S, 15.7; Found: C, 58.7; H, 4.15; N, 17.23; S, 15.63; IR (ATR, cm^−1^): 3093 (ν NH_triazole_), 1628 (ν -N=CH-), 1272 (ν C=S); 1254 (ν C-O), 1038 (ν C-O); ^1^H-NMR (500 MHz, DMSO-*d*_6_, *δ*/ppm): 13.95 (s, 1H, NH), 9.45 (s, 1H, -N=CH-), 7.92–8.05 (d, 2H, ArH), 7.71 (d, 1H, ArH), 7.47–7.55 (m, 4H, ArH), 7.29 (d, 1H, ArH), 7.09 (m, 1H, ArH), 3.86 (s, 3H, OCH_3_), 2.41 (s, 3H, CH_3_); ^13^C-NMR (125 MHz, DMSO-*d*_6_, *δ*/ppm): 169.97 (C=S), 159.10 (C), 157.88 (CH=N), 157.43 (C), 153.40 (C), 151.17 (C), 143.43 (C), 132.10 (CH), 131.81 (CH), 130.79 (2CH), 129.21 (2CH), 128.93 (CH), 128.72 (C), 121.39 (CH), 117.02 (C), 111.22 (CH), 55.69 (CH_3_), 15.91 (CH_3_); MS (EI, 70 eV) *m*/*z* (%): 408.49 (M + 1).

*4-(3-Methoxybenzylideneamino)-5-(4-methyl-2-phenylthiazol-5-yl)-4H-1,2,4-triazole-3-thiol* (**B13**). Yield 80.6% (0.680 g); m.p. 270 °C; yellow powder; Anal. Calcd for C_20_H_17_N_5_OS_2_ (407.51): C, 58.89; H, 4.17; N, 17.17; S, 15.7; Found: C, 58.7; H, 4.18; N, 17.23; S, 15.63; IR (ATR, cm^−1^): 3089 (ν NH_triazole_), 1621 (ν -N=CH-), 1283 (ν C=S); 1257 (ν C-O), 1034 (ν C-O); ^1^H-NMR (500 MHz, DMSO-*d*_6_, *δ*/ppm): 13.91 (s, 1H, NH), 9.44 (s, 1H, -N=CH-), 7.99–8.05 (d, 2H, ArH), 7.58 (s, 1H, ArH), 7.38–7.52 (m, 4H, ArH), 7.38 (d, 1H, ArH), 7.14 (d, 1H, ArH), 3.93 (s, 3H, OCH_3_), 2.43 (s, 3H, CH_3_); ^13^C-NMR (125 MHz, DMSO-*d*_6_, *δ*/ppm): 169.95 (C=S), 160.75 (C), 159.14 (C), 157.88 (CH=N), 153.37 (C), 151.09 (C), 143.41 (C), 134.80 (C), 130.85 (2CH), 129.86 (CH), 129.25 (2CH), 128.83 (CH), 128.66 (C), 121.62 (CH), 116.66 (CH), 111.28 (CH), 55.75 (CH_3_), 15.89 (CH_3_); MS (EI, 70 eV) *m*/*z* (%): 408.49 (M + 1).

*5-(4-Methyl-2-phenylthiazol-5-yl)-4-(thiophen-2-ylmethyleneamino)-4H-1,2,4-triazole- 3-thiol* (**B14**). Yield 79.3% (0.536 g); m.p. 280 °C; yellow powder; Anal. Calcd for C_17_H_13_N_5_S_3_ (383.51): C, 53.19; H, 3.38; N, 18.25; S, 25.03; Found: C, 53.4; H, 3.38; N, 18.32; S, 24.92; IR (ATR, cm^−1^): 3088 (ν NH_triazole_), 1627 (ν -N=CH-), 1288 (ν C=S); ^1^H-NMR (500 MHz, DMSO-*d*_6_, *δ*/ppm): 14.10 (s, 1H, NH), 9.46 (s, 1H, -N=CH-), 7.94–7.99 (d, 2H, ArH), 7.74–7.78 (d, 2H, ArH), 7.40–7.51 (m, 4H, ArH), 2.42 (s, 3H, CH_3_); ^13^C-NMR (125 MHz, DMSO-*d*_6_, *δ*/ppm): 170.08 (C=S), 159.13 (C), 157.95 (CH=N), 153.29 (C), 151.09 (C), 144.96 (C), 143.27 (C), 130.98 (2CH), 130.11 (CH), 129.27 (2CH), 128.91 (CH), 128.77 (C), 128.55 (CH), 127.30 (CH), 16.01 (CH_3_); MS (EI, 70 eV) *m*/*z* (%): 384.4 (M + 1).

*4-(4-(Dimethylamino)benzylideneamino)-5-(4-methyl-2 phenylthiazol-5-yl)-4H-1,2,4-triazole-3-thiol* (**B15**). Yield 77.3% (0.653 g); m.p. 260 °C; Anal. Calcd for C_21_H_20_N_6_S_2_ (420.55): C, 59.92; H, 4.75; N, 19.97; S, 15.21; Found: C, 59.7; H, 4.76; N, 19.89; S, 15.27; yellow powder; IR (ATR, cm^−1^): 3086 (ν NH_triazole_), 1619 (ν -N=CH-), 1331 (ν C-N), 1258 (ν C=S); ^1^H-NMR (500 MHz, DMSO-*d*_6_, *δ*/ppm): 14.01 (s, 1H, NH), 9.51 (s, 1H, -N=CH-), 7.96–7.98 (d, 2H, ArH), 7.45–7.53 (m, 3H, ArH), 6.99–7.34 (d, 4H, ArH), 3.31 (s, 6H, 2CH_3_), 2.47 (s, 3H, CH_3_); ^13^C-NMR (125 MHz, DMSO-*d*_6_, *δ*/ppm): 170.03 (C=S), 159.09 (C), 157.63 (CH=N), 153.53 (C), 153.28 (C), 151.06 (C), 138.27 (C), 130.97 (2CH), 129.33 (2CH), 128.81 (CH), 128.37 (2CH), 127.94 (C), 121.86 (C), 111.99 (2CH), 43.31 (2CH_3_), 16.09 (CH_3_); MS (EI, 70 eV) *m*/*z* (%): 421.12 (M + 1).

### 3.2. Antifungal Activity Assay

#### 3.2.1. Determination of Inhibition Zone Diameters

The in vitro antifungal activity was determined using the cup-plate agar diffusion method according to the Clinical and Laboratory Standards Institute (CLSI) guidelines [28]. For antifungal testing, Mueller-Hinton medium supplemented with 2% glucose (providing adequate growth of yeasts) and 0.5 mg/mL methylene blue (providing a better definition of the inhibition zone diameter) was used. The inoculum was prepared by suspending five representative colonies, obtained from an 18–24 h culture on non-selective nutritive agar medium, in sterile distilled water. The cell density was adjusted to the density of a 0.5 McFarland standard by measuring the absorbance in a spectrophotometer at a wavelength of 530 nm and adding sterile distilled water as required (corresponding to a population of 1–5 × 10^6^ CFU/mL). A sterile swab was soaked in suspension and then the Mueller-Hinton agar plates were inoculated by streaking the entire surface. After drying for 10–15 min, six-millimeter diameter wells were cut from the agar using a sterile cork-borer, and a volume of 20 μL of each compound solution (5 mg/mL in dimethyl sulfoxide-DMSO) was delivered into the wells (100 µg/well).

Fluconazole (100 μg/well) was used as the standard drugs. The controls were performed with only sterile broth, overnight culture and 20 μL of DMSO. The plates were incubated at 35 °C. Zone diameters were measured to the nearest whole millimeter at a point in which there will be no visible growth after 24–48 h. Results were obtained in triplicate. The solvent used for the preparation of each compound stock solution (5 mg/mL), DMSO (Merck, Darmstadt, Germany) exhibited no inhibitory activity against the tested fungal strain.

#### 3.2.2. Determination of MIC and MFC Values

The microorganisms used for antifungal activity evaluation were obtained from the University of Agricultural Sciences and Veterinary Medicine Cluj-Napoca Romania. The antifungal activity was evaluated against cultures of *Candida albicans* ATCC 10231, *Candida albicans* ATCC 18804, *Candida krusei* ATCC 6258. The cultures were stored on potato dextrose agar (Sifin, Germany). Prior to antifungal susceptibility testing, each strain was inoculated on potato dextrose agar plated to ensure optical growth characteristics and purity. Then, yeast cells were suspended in saline and adjusted spectrophotometrically to RPMI (Roswell Park Memorial Institute) 1640 medium to a final concentration of 10^6^ CFU/mL.

Stock solutions (1 mg/mL) were prepared by dissolving the test compounds, the reference antifungals (fluconazole and ketoconazole) in sterile DMSO. These solutions were stored at 4 °C. A series of double diluting solutions of the above compounds were prepared in RPMI 1640 medium obtaining final concentrations in the range of 500 µg/mL to 0.015 µg/mL. The broth microdilution method was employed for the minimum inhibitory concentration test. Media were placed into each of the 96 wells of the microplates. Sample solutions at high concentration (100 µg/mL) were added into the first rows of the microplates and two-fold dilutions of the compounds were made by dispensing the solutions into the remaining wells. Culture suspensions of 10 µL were inoculated into all the wells. The sealed microplates were incubated at 37 °C for 18 h. Antifungal activity was tested by using the broth microdilution method according to the Clinical and Laboratory Standard Institute (CLSI) guidelines [28]. The medium used for susceptibility testing was nutrient broth for bacteria and RPMI 1640 with l-glutamine, to pH 7.0 with 3-(*n*-morpholino)propanesulfonic acid. The initial density of *E. coli* and *S. aureus* was 10^5^ CFU/mL and *Candida sp* was approximately 2 × 10^6^ colony forming units (CFU)/mL. Inoculums (density of 0.5 in McFarland scale) were prepared in a sterile solution of 0.9% NaCl solution. Then, tested strains were suspended in nutrient broth and RPMI 1640 media to give a final density of 2 × 10^5^ CFU/mL. Solutions of the test compounds and suspensions of bacteria and fungi were inoculated onto 96-well microplates. The growth control, sterility control and control of antibacterial/antifungal compounds were used. Plates were incubated under normal atmospheric conditions at 30 °C for 48 h (*Candida albicans* ATCC 10231, *Candida albicans* ATCC 18804, *Candida krusei* ATCC 6258), and next minimum inhibitory concentration (MIC) values have been determined by adding resazurin (20 μL, 0.02%) followed by incubation for 2 h. The MIC was defined as the lowest concentration required to arrest the growth of the bacteria/fungi. For determination of minimum fungicidal concentration (MFC), a 0.01 mL aliquot of the medium drawn from the culture tubes showing no macroscopic growth at the end of the 24 h culture was subcultured on nutrient agar/potato dextrose agar plates to determine the number of vital organisms and incubated further at 30 °C for 48 h. The MFC was defined as the lowest concentration of the agent at which no colonies are observed. All MIC and MFC experiments were repeated three times.

### 3.3. Molecular Docking

A molecular docking study was carried out using AutoDock 4.2 [29], in order to investigate the possible interactions with cytochrome P450 14α-sterol demethylase (CYP51) from *Saccharomyces cerevisiae* and to predict the binding mode of our synthesized compounds. The crystallographic structure of the complex between the three-dimensional (3D) structure of the enzyme, with the intact transmembrane domain, and co-crystallized itraconazole (PDB:5EQB), was used for the docking study. The crystal structure of the enzyme obtained by X-ray diffraction was taken from Protein Data Bank [30].

A dataset of 3D thiazolyl-triazole Schiff bases **B1**–**B15** was generated using HyperChem 6, with geometry optimization and minimized energy conformation. Further, all input files were prepared using AutoDock Tools 1.5.6 [29]. Ligand preparation included addition of Gasteiger charges and merging non-polar hydrogen atoms. Fluconazole, a drug known to inhibit cytochrome P450 lanosterol 14α-demethylase [31], was taken as the positive control.

Using AutoDock Tools, co-crystallized ligand (itraconazole) and water were removed from the active site of the enzyme, polar hydrogen atoms were added, non-polar hydrogen atoms merged, rotatable bonds were defined, amide bonds were set non-rotatable, carboxylic moieties deprotonated and Gasteiger partial charges were assigned. The grid box was defined as *x* = *y* = *z* = 28 Å in dimension with *x* = 19.692, *y* = 14.168, *z* = 16.077 center coordinates. Grid maps were generated using AutoGrid 4 with 0.375 grid points size space.

AutoDock searched for the best conformers poses, based on the Lamarkian genetic algorithm. For each compound, AutoDock searched for 10 conformers. The maximum number of evaluation was set at 9 × 10^4^ , rate of mutation at 0.02, rate of crossover at 0.8, step size for translations at 2 Å and cluster tolerance at 2 Å.

### 3.4. ADME and Molecular Property Prediction

In this present investigation, the new thiazolyl-triazole Schiff bases were subjected to a theoretical in silico ADME and toxicity prediction study, under Lipinski’s “Rule of five” [32]. Lipinski’s parameters were calculated using web tool Swiss ADME [33] We have also assessed tPSA (topological polar surface area), a descriptor that allows prediction of bioavailability and the transport of an active compound by the blood–brain barrier [23]. Bioavailability is highly multifactorial, but is primarily driven by gastrointestinal absorption [34]. The percentage of the absorption was calculated according to the equation: %ABS = 109 − 0.345 × TPSA [35]. Also, water solubility, CYP2D6, CYP2D9, P-glycoprotein inhibition and phospholipidosis (PLD) induction were predicted.

## 4. Conclusions

Synthesis of Schiff bases **B1**–**B15** followed several steps. The 4-amino-5-(4-methyl-2-phenylthiazol-5-yl)-4*H*-1,2,4-triazole-3 thiol **4** was obtained by treating potassium 2-(4-methyl-2-phenylthiazole-5-carbonyl)hydrazine-carbodithioate **3**, previously obtained, with hydrazine hydrate. The final derivatives were synthesized in good yields by the condensation of compound **4** with various aromatic or heteroaromatic aldehydes.

Schiff bases were investigated for their anti-*Candida* potential. The in vitro methods used were the disk diffusion disk, the determination of MIC and MFC, respectively. The activity of the new derivatives was reported to reference drugs, already used in therapy. Some of the Schiff bases showed MICs inferior to those of the references used. Compound **B10** showed to be the most promising anti-*Candida* candidate, inhibiting the growth of all three *Candida* strains used and being more potent than fluconazole. The obtained results suggest that the new series bearing thiazole and triazole scaffolds may be considered for further investigation and optimization, in designing anti-*Candida* drugs.

A docking study of the thiazolyl-triazole Schiff bases was performed on compounds **B1**–**15** and presented a rationale for the activity of these compounds. Performed on CYP51 enzyme, this study showed that the thiazolyl-triazole Schiff bases interact with the amino acids in the access channel to the active site of the lanosterol 14α-demethylase with higher binding affinity energy than fluconazole. Moreover, the compounds do not interact with the heme.

Furthermore, a virtual study was performed to predict the ADME and the toxicity of the derivatives. The studied compounds showed an acceptable gastro-intestinal absorption and a low to moderate water solubility. We could predict the absence of toxicity at CNS level, due to the fact that the compounds do not pass the blood–brain barrier. All of the newly synthesized compounds were predicted as noninhibitors of CYP2D6, noninducers of phospholipidosis, and they are not a substrate for P-glycoprotein. All these observations will be helpful in designing newer anti-*Candida* agents with good pharmacokinetic properties and without severe side effects. 

## Figures and Tables

**Table 1:** The inhibition zone diameters on *Candida albicans*.

Cp	Inhibition Zone Diameter (mm)
	*Candida albicans* ATCC 90028
**B1**	16
**B2**	18
**B3**	18
**B4**	18
**B5**	**20**
**B6**	18
**B7**	18
**B8**	18
**B9**	18
**B10**	**20**
**B11**	**20**
**B12**	18
**B13**	18
**B14**	18
**B15**	18
**Fluconazole**	**25**

The values obtained for the most active compounds are marked in bold.

**Table 2:** Minimum Inhibitory Concentration—MIC (in μg/mL) of compounds **B1**–**B15**.

Cp	*C. albicans* ATCC 10231	*C. albicans* ATCC 18804	*C. krusei* ATCC 6258
**B1**	62.5	62.5	62.5
**B2**	62.5	62.5	62.5
**B3**	62.5	62.5	62.5
**B4**	62.5	62.5	62.5
**B5**	62.5	62.5	**31.25**
**B6**	62.5	62.5	62.5
**B7**	62.5	62.5	62.5
**B8**	62.5	**31.25**	62.5
**B9**	62.5	62.5	62.5
**B10**	**15.62**	**31.25**	**31.25**
**B11**	62.5	62.5	62.5
**B12**	62.5	62.5	62.5
**B13**	62.5	62.5	62.5
**B14**	62.5	**31.25**	62.5
**B15**	62.5	62.5	62.5
**Fluconazole**	**62.5**	**62.5**	**62.5**
**Ketoconazole**	**31.25**	**31.25**	**31.25**
**Inoculum control**	+++	+++	+++
**Broth control**	No growth	No growth	No growth

+++ indicates growth in all concentrations; the values obtained for the most active compounds are marked in bold.

**Table 3:** Minimum Fungicidal Concentration—MFC (in μg/mL) of compounds **B1**–**B15**.

Cp.	*C. albicans* ATCC 10231	*C. albicans* ATCC 18804	*C. krusei* ATCC 6258
**B1**	125	125	125
**B2**	125	125	125
**B3**	125	125	125
**B4**	125	125	125
**B5**	125	125	**62.5**
**B6**	125	125	125
**B7**	125	125	125
**B8**	125	**62.5**	125
**B9**	125	**62.5**	125
**B10**	**31.25**	**62.5**	**62.5**
**B11**	125	125	125
**B12**	125	125	125
**B13**	125	125	125
**B14**	125	**62.5**	125
**B15**	125	125	**62.5**
**Fluconazole**	**125**	**125**	**125**
**Ketoconazole**	**62.5**	**62.5**	**62.5**

The values obtained for the most active compounds are marked in bold.

**Table 4:**
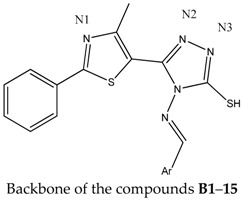
Predicted binding affinity and polar contacts between compounds **B1**–**B15** and the active site of CYP51.

Samples	Binding Affinity (kcal/mol)	Atom ID of Ligand	Interacting AA Residue	Bond Length (Å)
**B1**	−11.39	N1	Met509	3.0
		N2	Ser508	3.5
		N3	Phe506	2.6
**B2**	−11.85	N2	Ser508	3.5
		N3	Phe506	2.6
**B3**	−11.59	N1	Met509	2.9
		N2	Ser508	4.4
		N3	Phe506	2.6
**B4**	−11.08	N1	Ser382	3.1
		N2	Ser508	2.9
		N3	Ser508	3.2
		N3	Phe506	2.5
**B5**	−11.22	N1	Met509	3.2
		N2	Ser508	2.9
		N3	Phe506	2.5
**B6**	−10.30	N1	Met509	2.9
		N2	Phe506	2.7
		N3	Phe506	2.7
**B7**	−10.92	N1	Met509	2.9
		N2	Ser508	3.6
		N3	Phe506	2.6
**B8**	−11.25	N1	Ser382	3.1
		N2	Phe506	3.0
		N3	Phe506	2.7
		Phenolic O	Arg98	3.1
**B9**	−10.84	N1	Ser382	3.0
		N2	Ser508	2.9
		N3	Ser508	3.2
		N3	Phe506	2.6
**B10**	−12.92	N1	Met509	2.6
		N2	Ser508	3.3
		N2	Phe506	2.5
		N3	Phe506	2.5
		Nitro O	Arg98	3.0
		Nitro O	Arg98	3.2
		Nitro O	Leu95	2.7
**B11**	−11.70	N1	Met509	2.9
		N2	Ser508	3.5
		N3	Phe506	2.6
		Nitro O	Arg98	3.4
**B12**	−11.17	N1	Ser382	2.8
		N2	Ser508	2.9
		N3	Ser508	3.0
		N3	Phe506	2.5
**B13**	−10.93	N1	Ser382	3.3
		N2	Ser508	3.2
		N3	Phe506	2.8
**B14**	−10.45	N1	Ser382	3.1
		N2	Ser508	2.8
		N3	Ser508	3.2
		N3	Phe506	2.6
**B15**	−11.18	N1	Met509	2.8
		N2	Ser508	3.4
		N3	Phe506	2.6
**Fluconazole**	−7.03	N/A	N/A	N/A

**Table 5:** Virtual ADME (absorption, distribution, metabolism, excretion) and molecular property prediction of the Schiff bases **B1**–**B15**.

Comp.	tPSA ^a^	%Abs ^b^	MW ^c^	RoB ^d^	HBD ^e^	HBA ^f^	MR ^g^	IlogP ^h^ (MlogP)	LogS ^i^	CYP2D6 Inhibitor
**Rule**	≤140 Ǻ^2^	-	≤1500	≤110	≤15	≤110	40–130	<5	>−4	
**B1**	123	66.57	446.38	4	0	4	118.24	3.77 (4.12)	−8.19	No
**B2**	123	66.57	446.38	4	0	4	118.24	3.69 (4.12)	−8.19	No
**B3**	123	66.57	446.38	4	0	4	118.24	3.42 (4.12)	−8.19	No
**B4**	123	66.57	411.93	4	0	4	113.23	3.86 (3.89)	−7.60	No
**B5**	123	66.57	456.38	4	0	4	115.92	3.35 (4.01)	−7.80	No
**B6**	123	66.57	395.48	4	0	5	108.18	3.3 (3.78)	−7.28	No
**B7**	143.23	59.59	393.49	4	1	5	110.24	3.09 (2.85)	−6.43	No
**B8**	143.23	59.59	393.49	4	1	5	110.24	3.12 (2.85)	−6.43	No
**B9**	143.23	59.59	393.49	4	1	5	110.24	3.24 (2.85)	−6.43	No
**B10**	168.82	50.76	422.48	5	0	6	117.04	2.78 (2.42)	−6.84	No
**B11**	168.82	50.76	422.48	5	0	6	117.04	2.75 (2.42)	−6.84	No
**B12**	132.23	63.38	407.51	5	0	5	114.71	3.98 (3.08)	−7.12	No
**B13**	132.23	63.38	407.51	5	0	5	114.71	3.84 (3.08)	−7.12	No
**B14**	151.24	56.82	383.51	4	0	4	106.09	3.26 (2.96)	−6.28	No
**B15**	126.24	65.45	420.55	5	0	4	122.42	3.82 (3.3)	−7.09	No

Abbreviations: ^a^ Topological polar surface area; ^b^ Absorption; ^c^ Molecular weight; ^d^ Number of rotatable bonds; ^e^ Number of hydrogen bond donors; ^f^ Number of hydrogen bonds acceptors; ^g^ Molar refractivity; ^h^ Logarithm of compound partition coefficient between *n*-octanol and water; ^i^ Logarithm of water solubility.
